# Establishing a robotic-assisted PCI program: experiences at a large tertiary referral center

**DOI:** 10.1007/s00380-022-02078-z

**Published:** 2022-04-29

**Authors:** Fabian J. Brunner, Christoph Waldeyer, Elvin Zengin-Sahm, Christoph Kondziella, Benedikt Schrage, Peter Clemmensen, Dirk Westermann, Stefan Blankenberg, Moritz Seiffert

**Affiliations:** 1 Department of Cardiology, University Heart & Vascular Center Hamburg, University Medical Center Hamburg-Eppendorf, Martinistrasse 52, 20246 Hamburg, Germany; 2 German Center of Cardiovascular Research (DZHK), Partner Site Hamburg/Kiel/Lübeck, Hamburg, Germany

**Keywords:** Calcified stenosis, Chronic coronary total occlusion, Other technique, Multiple vessel disease, Robotic PCI

## Abstract

Robotic-assisted percutaneous coronary interventions (rPCI) have proven feasible and safe while reducing radiation exposure for the operator. Recently, rPCI systems have been refined to facilitate the treatment of complex lesions. The aim of the current study was to evaluate challenges and opportunities of establishing an rPCI program at a tertiary referral center. rPCI was performed using the CorPath GRX Vascular Robotic System (Corindus Inc., a Siemens Healthineers Company, Waltham, USA). Baseline, procedural, and in-hospital follow-up data were prospectively assessed. rPCI success was defined as completion of the PCI without or with partial manual assistance. The safety endpoint was the composite of missing angiographic success or procedure-related adverse events during hospital stay. Overall, 86 coronary lesions were treated in 71 patients (28.2% female) from January to April 2021. Median age was 71.0 years (IQR 60.3; 79.8). Indications for rPCI were stable angina pectoris (71.8%), unstable angina (12.7%) and non-ST elevation myocardial infarction (15.5%). Most lesions were complex (type B2/C: 88.4%) and included 7 cases of rPCI for chronic total occlusions. Angiographic and rPCI success were achieved in 100.0% and 94.2%, respectively. Partial manual assistance was used in 25.6%. Conversion to manual PCI was required in 5.8%. The safety endpoint occurred in 7.0% of patients. rPCI when applied as clinical routine for complex coronary lesions is effective with good immediate angiographic and clinical results. Future investigations should focus on the identification of patients that particularly benefit from robotic-assisted vs. manual PCI despite higher resource utilization.

## Introduction

Percutaneous coronary interventions (PCIs) are routinely performed for the treatment of patients with acute or chronic coronary syndromes [1]. Despite all measures of radiation protection [2], operators and patients are exposed to a significant amount of radiation, particularly during complex procedures, with their inherent harms [3–5]. Additional lead protection may reduce radiation exposure, however, at the cost of increased musculoskeletal injuries [6, 7]. Robotic-assisted PCI (rPCI) systems have been developed [8], reducing some of these occupational hazards associated with PCI while demonstrating feasibility and safety [9–12]. Additional resources, costs, and technical limitations of the early rPCI systems have limited broad dissemination among interventional cardiologists. Recently, rPCI platforms have been refined to facilitate the treatment of complex lesions [13]. However, insights on their use in clinical routine remain limited. We, therefore, aimed to evaluate challenges and opportunities of establishing a latest generation rPCI program at our institution and report results of 71 consecutive rPCI procedures.

## Methods

### The rPCI system

**Fig. 1:**
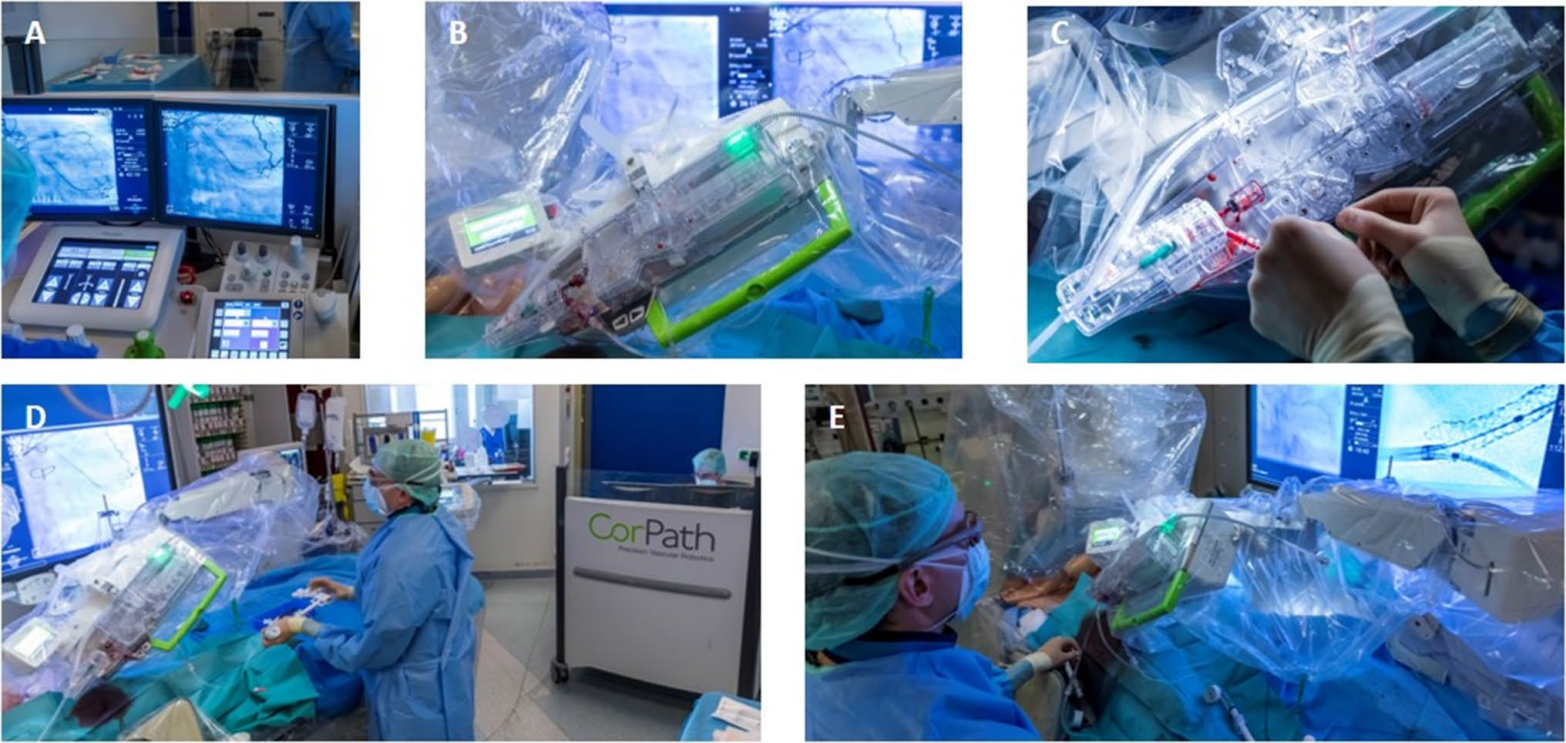
The rPCI platform and setup in the catheterization laboratory. The interventionalist’s cockpit features remote controls for the rPCI system, c -arm, X-ray tube, and radiation protection shields (**A**). The robotic arm, housing the disposable cassette, after setup and connection to the sheath and guiding catheter enabled for robotic-assisted navigation (**B**). Exchange of wires and devices by the assistant is performed at the patient’s bedside (**C**). The setup allowed for a distance between the assistant and the c-arm to reduce X-ray exposure (**D**). The “hybrid approach” was utilized for bifurcation stenting to accelerate the procedure: the second balloon or stent was guided manually by the second operator while the first device was navigated robotic-assisted (**E**)

**Fig. 2:**
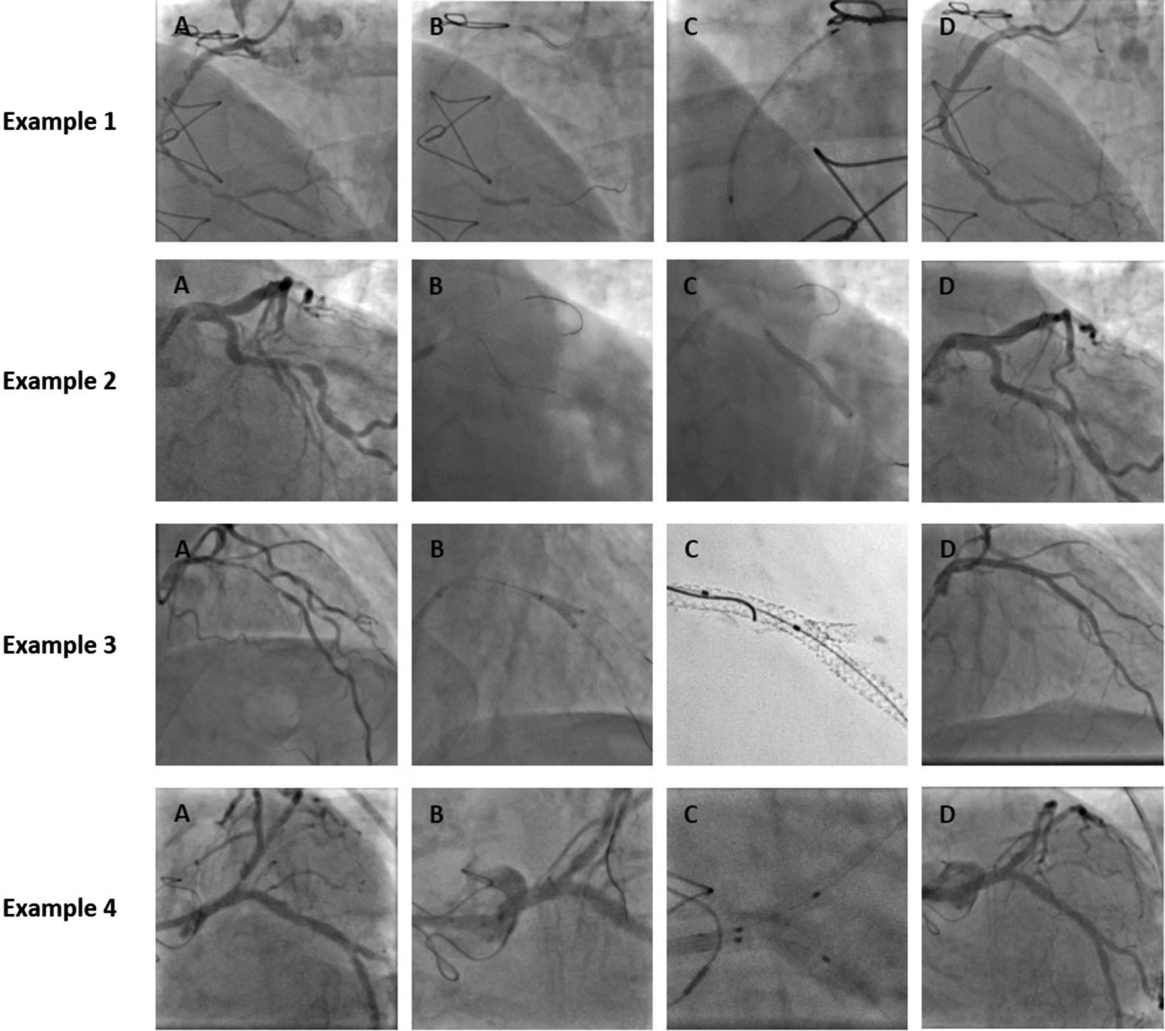
Coronary angiograms of complex robotic-assisted procedures. *Example 1* illustrates the rPCI of a long segment stenosis of the right coronary artery (**A**). The complete procedure was performed robotic-assisted (**B**–**D**). *Example 2* demonstrates a severe stenosis of a tortuous circumflex artery (CFX) (**A**). Navigation of a stabilization wire into the LAD and lesion crossing in the CFX, pre-dilatation and stent deployment was performed robotic-assisted (**B**–**C**). Final result as depicted in (**D)**. *Example 3* shows the treatment of a bifurcation stenosis of the LAD/diagonal branch with the double-kissing crush technique (**A**). A “hybrid approach” was used to guide two devices simultaneously by a combination of manual and robotic-assisted movements, for example of the final kissing-balloon maneuver (**B**). High-resolution imaging and final angiogram demonstrated an excellent result (**C**–**D**). The patient in *example 4* had a history of PCI of the left main stem (LM) and the proximal CFX (provisional stent implantation) and underwent angiogram due to unstable angina pectoris. Two de novo stenoses of the ostial LM and the LAD were observed (**A**). After pre-dilatation, a 28 mm drug-eluting stent was implanted from the ostial LM into the proximal LAD (**B**). The final kissing-balloon maneuver was performed as a “hybrid approach” as described before (**C**) achieving an excellent result (**D**)

rPCI was performed using the 2nd generation CorPath GRX Vascular Robotic System (Corindus Inc., a Siemens Healthineers Company, Waltham, USA) with the Artis zee floor platform (Siemens Healthineers AG, Erlangen, Germany). The CorPath GRX platform consisted of an interventional cockpit and a robotic arm (Fig. 1A–E). The cockpit was located within the cardiac catheterization laboratory and featured controls for the c-arm, the X-ray tube, and the robotic arm. Radiation protection shields facilitated lead-free working near a high-resolution monitor (Fig. 1A). The workstation featured three joysticks for guide catheter manipulation, steering of the guidewire and driving of devices (e.g. balloon and stent catheters). The CorPath GRX included novel automated movements (technIQ™) to improve guidewire navigation: “Wiggle” (oscillation of the wire during advancement to prevent prolapse), “Spin” (clockwise and counterclockwise rotation to enhance lesion crossing), “Rotate-on-Retract” (automatic clockwise rotation of the guidewire during retraction to redirect the tip; RoR), “Dotter” (rapid linear back-and-forth motions of the delivery system to ease lesion crossing) and “Constant Speed” to navigate guide wires or catheters at a consistent speed (2 mm/s and 5 mm/s). Exemplary cases demonstrating the advanced guidewire navigation are provided in Fig. 2.

The robotic arm was mounted to the patients’ bedside and housed the sterile cassette (Fig. 1B). The radial or femoral sheaths and guide catheters were attached to these cassettes after engagements of the coronary ostia to enable robotic-assisted navigation (Fig. 1B). The assistant was scrubbed in at the bedside, setting up the robotic arm, feeding the system with guide wires and delivery systems, and inject contrast (Fig. 1C–E). The first operator navigated the procedure from the interventional cockpit (Fig. 1A). During the setup of our program, two interventional cardiologists were trained for the rPCI system and alternated in performing the procedures as first operator or assistant. In addition, seven technicians were trained during the study period to set up the rPCI system and to provide technical assistance during the procedure.

### Study population

From January to April 2021, in 71 patients, rPCI procedures were performed at the University Heart and Vascular Center in Hamburg, Germany. Indication for PCI and periprocedural care, including antithrombotic regimens, were based on current guidelines [1]. Allocation of patients to rPCI was based on clinical decisions and availability of trained staff. Preferably, in patients scheduled for elective rPCI, the initial setup of the platform was done before the vascular access was gained. In some cases, ad hoc rPCI was performed immediately after diagnostic angiography for acute coronary syndrome. Patients eligible for rPCI procedure were enrolled in the prospective cohort study INTERCATH (NCT04936438, please see [14, 15] for more details). Routine clinical, laboratory and procedural parameters were documented after written informed consent was obtained. The study protocol of the INTERCATH study was approved by the Hamburg Ethics Committee.

### Data management and endpoint definition

Baseline, procedural, and follow-up data were prospectively assessed and analyzed. Offline quantitative coronary angiography was performed from digitally recorded coronary angiograms. Adapted from the literature, rPCI success was defined as completion of the PCI without manual assistance or with partial manual assistance, planned or unplanned, with ultimate completion of the procedure robotically. Complete manual conversion was defined as the need for bedside manipulation of either the guide catheter, guidewire, or delivery system, which was required to complete the procedure [11]. Angiographic success was defined as a residual diameter stenosis < 20% of the target lesion and Thrombolysis in Myocardial Infarction (TIMI) 3 flow. The safety endpoint was the composite of missing angiographic success or procedure-related adverse events within 72 h after the procedure or before hospital discharge, whichever occurred fist. Procedure-related adverse events were defined as follows: All-cause death, procedural myocardial infarction, stroke, and ischemia-driven target lesion revascularization. Procedural myocardial infarction was defined according to the Society for Cardiovascular Angiography and Interventions (SCAI) recommendations [16].

## Results

### Study population

During the study period, a total of 71 patients were treated with the rPCI system. Median age was 71.0 years (interquartile range; IQR 60.3; 79.8) and 28.2% were female. The cardiovascular risk profile and baseline characteristics are listed in Table 1. Indication for rPCI was stable angina pectoris and relevant ischemia in 71.8%, unstable angina in 12.7% and non-ST elevation myocardial infarction in 15.5% of patients. Patients reported angina pectoris CCS (Canadian Cardiovascular Society) classes III or IV in 39.4% and New York Heart Association (NYHA) classes III or IV in 52.1%. Overall, a total of 86 coronary lesions were treated (1.2 ± 0.4 lesions per patient). Most lesions were complex (type B2: 18.6%, type C: 69.8%) and included 7 cases of PCI for chronic total occlusions (CTO), accounting for 8.1% of the study population (Table 2).

**Table 1:** Baseline characteristics

	*N* = 71 patients
Age, years (IQR)	71.0 (60.3; 79.8)
Female, %	28.2
Body-mass-index, kg/m^2^ (IQR)	26.3 (24.2; 29.1)
Arterial hypertension, %	85.9
Diabetes mellitus, %	38.0
Current smoking, %	19.7
History of smoking, %	39.4
Left-ventricular EF < 40%, %	28.2
EF, % (IQR)	50.0 (36.5; 56.5)
Laboratory parameters	
LDL-C, mg/dL (IQR)	95.0 (69.3; 117.5)
Serum creatinine, mg/dL (IQR)	1.0 (0.8; 1.4)
eGFR, ml/min/1.73m^2^ (IQR)	69.0 (45.5; 82.5)
CK, U/L (IQR)	77.0 (48.3; 110.8)
hsTnI, ng/L (IQR)	18.0 (9.0; 59.5)
History of cardiovascular disease	
* Cerebrovascular disease, %*	16.9
* PAD, %*	16.9
* S/p PCI, %*	59.2
* S/p CABG, %*	14.1
S/p myocardial infarction, %	39.4
Clinical presentation	
* SAP, %*	71.8
* UAP, %*	12.7
* NSTEMI, %*	15.5
Symptoms	
* CCS classification, %*	
* I*	26.8
* II*	33.8
* III*	32.4
* IV*	7.0
* NYHA classification, %*	
* I*	1.4
* II*	46.5
* III*	49.3
* IV*	2.8

Data are shown as relative numbers or median (interquartile range; IQR)

*EF* ejection fraction, *LDL-C* cholesterol related to low-density lipoproteins, *eGFR* estimated glomerular filtration rate, *CK* creatinine kinase, *hsTnI* high-sensitivity cardiac troponin I, *PAD* peripheral artery disease, *PCI* percutaneous coronary intervention, *CABG* coronary artery bypass graft surgery, *SAP/UAP* stable/unstable angina pectoris, *NSTEMI* non-ST segment elevation myocardial infarction, *CCS* Canadian Cardiovascular Society, *NYHA* New York Heart Association

**Table 2:** Procedural aspects and follow-up

	*N* = 71 patients
Radial access site, %	73.2
Number of lesions treated, *N*	86
Robotically treated lesions per patient ± SD	1.2 ± 0.4
Treated lesion characteristics	
Type A, %	1.2
Type B1, %	10.5
Type B2, %	18.6
Type C, %	69.8
CTO, %	8.1
Left Main, %	4.7
Bifurcation, %	46.5
In-stent restenosis, %	9.3
Coronary artery bypass graft, %	2.3
In-hospital follow-up	
Serum creatinine, mg/dL (IQR)	1.0 (0.8; 1.4)
eGFR, mL/min/1.73m^2^ (IQR)	75.5 (43.5; 83.8)
CK, U/L (IQR)	74.0 (50.0; 106.0)
hsTnI, ng/L (IQR)	275.0 (76.8; 1,005.0)
Safety endpoint, %	7.0
Missing angiographic success	*N* = 0
All-cause death	*N* = 0
Procedural myocardial infarction (SCAI)	*N* = 4
Ischemia-driven TLR	*N* = 0
Non-disabling ischemic stroke	*N* = 1

Data are shown as absolute and relative numbers or median (interquartile range; IQR). Bifurcation was defined as any target lesion including an angiographically relevant sidebranch. The safety endpoint was defined as the composite of missing angiographic success or procedure-related adverse events (all-cause death, procedural myocardial infarction, stroke, and ischemia-driven target lesion revascularization). Procedural myocardial infarction was defined according to the Society for Cardiovascular Angiography and Interventions (SCAI) recommendations [16] and based on increased htTnI values; none of the patients featured clinical symptoms of ischemia

*CTO* chronic total occlusion, *eGFR* estimated glomerular filtration rate, *CK* creatinine kinase, *hsTnI* high-sensitivity cardiac troponin I, *TLR* target lesion revascularization

### Setup of the rPCI system

Most patients (84.5%) underwent scheduled rPCI and the initial setup of the CorPath GRX platform (see below for details) was performed during preparation and draping of the patient before vascular access was gained. In 15.5% of patients with unknown coronary status, ad hoc rPCI was performed immediately after diagnostic coronary angiography and the rPCI system was set up after coronary angiography had been completed. In these cases, the initial setup of the platform (adjusting and draping the robotic arm, insertion of the disposable cassette and activation of the system) was accelerated to a minimum of 3:20 min following training of the cathlab staff.

### Procedural aspects

rPCI was performed via radial access in 73.2% of patients (Table 2). Reasons to perform transfemoral rPCI were PCI for CTO, insufficient backup, or known previous unsuccessful radial access. Two cases were converted from radial to femoral due to insufficient length of the guiding catheter to provide an adequate connection to the robotic arm. Most target lesions were located in the left anterior descending artery (LAD; 39.5%) or right coronary artery (RCA; 33.7%). Other target lesions were located in the left circumflex artery (CFX; 23.3%), the left main stem (5.8%), or venous bypass grafts (2.3%) (Table 3). Moderate or severe calcifications were observed in 59.3% of lesions with increasing prevalence in type B2 (68.8%) and type C (67.9%) lesions (exemplary cases are shown in Fig. 2). Lesion length, number of implanted drug-eluting stents and procedure duration as well as dose area product increased with lesion complexity. Acute angiographic success was achieved in all cases (Table 3).

**Table 3:** Lesion characteristics and rPCI details

	Overall (*N* = 86)	Type A/B1 (*N* = 10)	Type B2 (*N* = 16)	Type C w/o CTO (*N* = 53)	CTO (*N* = 7)
Coronary artery treated					
Left main, *N* (%)	5 (5.8%)	0	0	4 (7.5%)	1 (14.3%)
LAD, *N* (%)	34 (39.5%)	2 (20.0%)	8 (50.0%)	22 (41.5%)	2 (28.6%)
CFX, *N* (%)	20 (23.3%)	2 (20.0%)	4 (25.0%)	11 (20.8%)	3 (42.9%)
RCA, *N* (%)	29 (33.7%)	6 (60.0)	4 (25.0%)	17 (32.1%)	2 (28.6%)
ACVB, *N* (%)	2 (2.3%)	0	0	2 (3.8%)	0
Calcification level					
None‚/mild, %	40.7	100.0	31.3	32.1	42.9
Moderate, %	37.2	0	50.0	41.5	28.6
Severe, %	22.1	0	18.8	26.4	28.6
IVUS imaging	11 (12.8%)	0	2 (12.5%)	5 (9.4%)	4 (57.1%)
Lesion length, mm (IQR)	24.0 (12.4; 39.3)	11.0 (8.0; 12.0)	12.4 (8.5; 18.1)	29.5 (22.1; 41.7)	52.9 (35.1; 74.6)
Reference diameter, mm (IQR)	3.0 (2.7; 3.4)	2.6 (2.5; 3.5)	2.9 (2.7; 3.3)	3.0 (2.8; 3.4)	2.9 (2.7; 3.0)
IVL preparation	3 (3.5%)	0	0	3 (5.7%)	0
Number of DES used ± SD	1.7 ± 0.9	1.0 ± 0	1.1 ± 0.3	1.8 ± 0.9	2.3 ± 1.4
Number of DCB used ± SD	0.1 ± 0.4	0	0	0.1 ± 0.1	0.6 ± 1.1
Stenosis before PCI, % (IQR)	79.2 (64.9; 92.0)	71.4 (65.0; 80.0)	76.9 (62.8; 83.7)	78.5 (64.8; 88.0)	100.0
Stenosis after PCI, % (IQR)	3.8 (2.8; 6.7)	3.8 (1.8; 4.0)	5.7 (3.4; 7.7)	3.7 (2.8; 6.5)	3.7 (1.9; 10.2)
Angiographic success rate, %	100.0	100.0	100.0	100.0	100.0
Robotic parameters					
Ready for use, min (IQR)	4.0 (3.1; 5.6)	2.9 (2.2; 5.0)	4.6 (4.0; 5.7)	3.6 (3.0; 5.6)	n/a
Wire crossing target lesion, min (IQR)	1.4 (0.8; 2.9)	1.5 (1.4; 2.3)	2.0 (0.9; 3.3)	1.3 (0.8; 2.8)	n/a
Balloon crossing target lesion, min (IQR)	1.0 (0.6; 1.8)	1.3 (0.7; 1.8)	0.8 (0.5; 2.1)	1.3 (0.7; 1.9)	n/a
Wire technIQ					
Wiggle, %	45.5	28.6	38.5	50.0	n/a
Spin, %	45.5	57.1	30.8	47.8	n/a
Rotate-on-retract, %	21.2	14.3	23.1	21.7	n/a
rPCI success, %	94.2	100.0	100.0	90.6	100.0
Manual assistance, %	25.6	10.0	18.8	20.8	100.0
Manual conversion, %	5.8	–	–	9.4	–
Total procedure time, min (IQR)	51.5 (33.6; 79.2)	26.7 (19.7; 28.8)	45.1 (29.8; 52.0)	54.5 (36.0; 78.1)	79.4 (77.8; 109.1)
Fluro time, min	20.4 (13.8; 27.8)	13.7 (11.2; 19.1)	19.3 (11.1; 26.1)	20.4 (14.9; 27.3)	29.3 (25.4; 44.1)
Dose-area-product, cGy*cm^2^	2,298 (1,626; 4,058)	995 (900; 2,611)	1,986 (1,681; 2,473)	2,298 (1,631; 3,738)	6,211 (5,892; 8,309)
Total contrast fluid, mL	145 (103; 188)	120 (82; 144)	136 (93; 157)	145 (107; 181)	199 (172; 233)

Data are shown as absolute and relative numbers, median (interquartile range; IQR), or mean ± standard deviation. Angiographic success was defined as a residual diameter stenosis < 20% of the target lesion and Thrombolysis in Myocardial Infarction (TIMI) 3 flow. Robotic parameters were defined as follows: *Ready for use*: time for connection of the robotic arm to the sheath and guiding catheter, shaping and insertion of a coronary guidewire and enabling the system for remote control; *Wire crossing target lesion*: time for guidewire crossing the target lesion after activation of remote control; *balloon crossing target lesion*: time for balloon crossing target lesion after advancing the guidewire. rPCI success was defined as completion of the PCI without manual assistance or with partial manual assistance, planned or unplanned, with ultimate completion of the procedure robotically. Complete manual conversion was defined as the need for bedside manipulation of either the guide catheter, guidewire, or delivery system, which was required to complete the procedure

*LAD* left anterior descending artery, *CFX* circumflex coronary artery, *RCA* right coronary artery, *ACVB* aortocoronary venous bypass graft, *IVUS* intravascular ultrasound, *IVL* intravascular lithotripsy, *DES* drug-eluting stent, *DCB* drug coated balloon, *PCI* percutaneous coronary intervention

### Specific aspects of rPCI

After the initial setup of the CorPath GRX platform, final preparation of the robotic arm (connection of the robotic arm to the sheath and guiding catheter, shaping and insertion of a coronary guidewire and enabling the system for remote control) required a median of 4.0 min (IQR 3.1; 5.6) (Table 3). Subsequent to activation of remote control, the coronary guidewire was advanced and crossed the target lesion in 1.4 min (IQR 0.8; 2.9). Automated wire movements (technIQ™) were used in the majority of cases to access the target: Wiggle (45.5%), Spin (45.5%), Rotate-on-retract (21.2%). The first balloon was delivered to the target lesion at a median of 1.0 min (IQR 0.6; 1.8). We did not observe differences in wire or balloon crossing time according to lesion complexity. However, procedural streamlining (e.g. pre-loading the coronary guidewire with a PCI balloon) was able to reduce the duration for balloon delivery after successful wire crossing down to a minimum of 15 s.

**Fig. 3:**
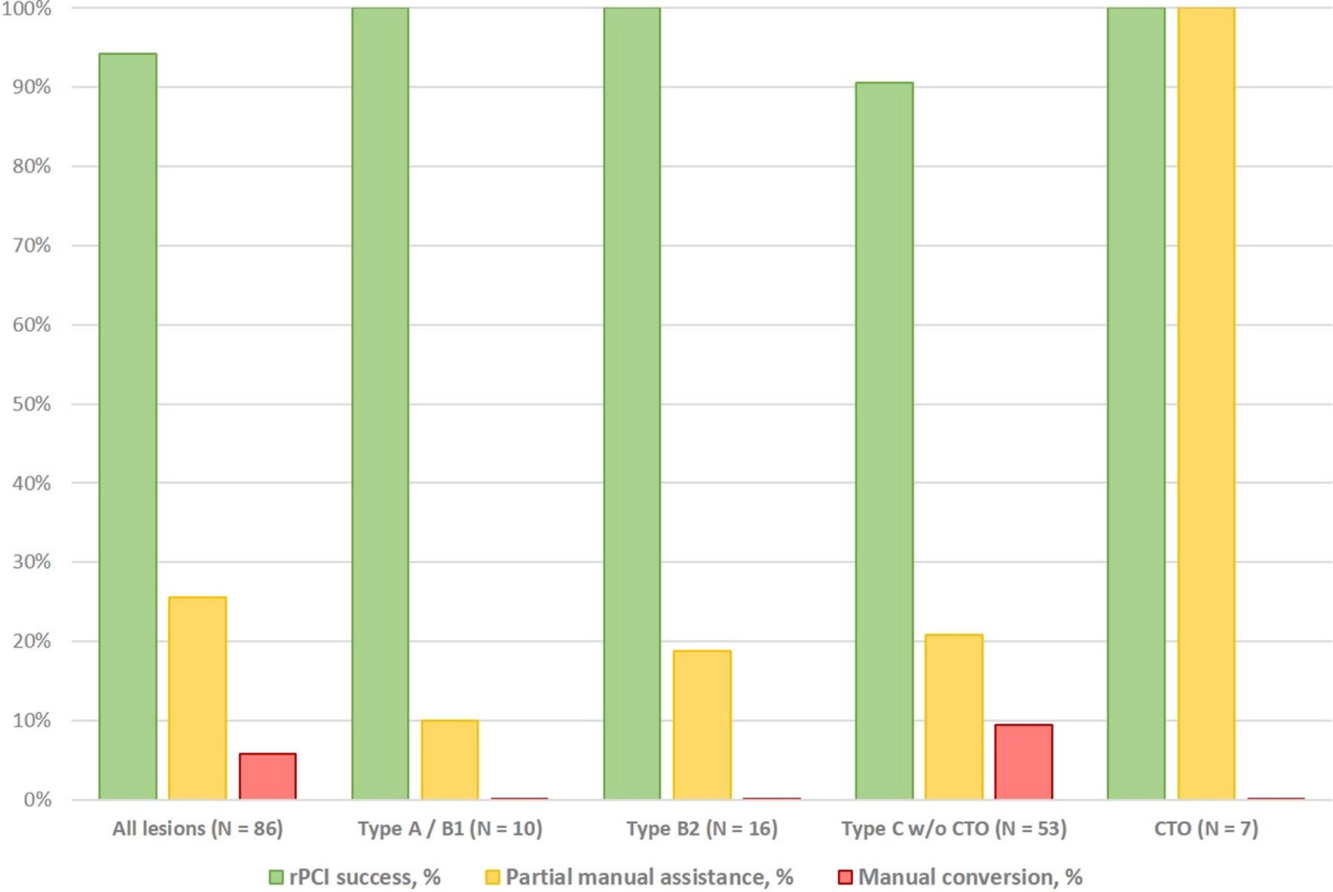
Rates of rPCI success, partial manual assistance and manual conversion according to lesion severity. Angiographic success was achieved in all cases. rPCI success was defined as completion of the PCI without manual assistance or with partial manual assistance (e.g. wire crossing in CTO lesions, “hybrid approach” in bifurcation stenting). Complete manual conversion was defined as the need for bedside manipulation of either the guide catheter, guidewire, or delivery system, which was required to complete the procedure. *CTO* chronic total occlusion

Angiographic and rPCI success were achieved in 100% and 94.2%, respectively. A total of 59 lesions (68.6%) were completely treated with rPCI. Manual assistance was performed in 25.6% of procedures (Fig. 3). All CTO PCI procedures (*N* = 7) were planned with manual assistance (Table 3). Initial crossing of the CTO body was performed manually with antegrade wire escalation techniques and dedicated CTO wires. After successful crossing and exchange for a workhorse wire over a microcatheter, the procedures were completed with rPCI in all cases. In addition, manual assistance was planned in 4 cases to complete bifurcation stenting and kissing-balloon maneuvers (Fig. 2). This “hybrid approach” allowed for navigation of two balloon catheters simultaneously, one with the CorPath GRX platform and one manually (Fig. 1E). Unplanned manual assistance was required due to insufficient backup of the guiding catheter, requiring initial manual wiring for stabilization (*N* = 2) or manual control to achieve optimal results in ostial lesions (*N* = 3). Partial manual support was required in 6 cases to navigate balloons or stents through to severe target vessel calcification or tortuosity. After manual assistance, including the use of intravascular lithotripsy in one case, these procedures were successfully completed with rPCI. Conversion to manual PCI was performed in 5 lesions (5.8%), mostly due to severe friction and the inability to advance any delivery system robotically or the repeated use of microcatheters. In one case, we observed a contained wire-induced side branch perforation in a patient with a tortuous target vessel that was managed conservatively without complications.

### Follow-up

The safety endpoint occurred in 5 patients (7.0%) that suffered from procedure-related adverse events (Table 2). Based on elevated high-sensitivity Troponin I blood levels, a periprocedural myocardial infarction was observed in 4 patients. However, none of these patients were symptomatic or had any further evidence of myocardial ischemia or infarction in the electrocardiogram nor by imaging. None of these patients had need for target lesion revascularization. One patient developed a non-disabling ischemic stroke after a complex rPCI of a venous bypass graft stenosis (type C lesion). This patient was discharged 5 days after the procedure without any residual neurologic deficits.

## Discussion

After successfully implementing a latest generation rPCI program, we evaluated the challenges, opportunities and the clinical outcomes when applied in the daily routine of a tertiary referral center finding that:

1. Setup of an rPCI program was easily feasible but additional prep time has to be considered.
2. rPCI procedures were safe and effective with high rates of success and low rates of conversion to manual PCI.
3. Latest refinements like the automated wire movements (technIQ™) were a valuable addition and a partial manual assistance (“hybrid approach”) proved helpful to support rPCI, particularly in complex lesions.

Establishing an rPCI program in clinical routine at a tertiary referral center was straightforward. In our experience, the learning curve for experienced interventional cardiologists was short. Only two to three rPCI procedures were sufficient to achieve a safe and quick handling of the robotic system. Moreover, the application of rPCI encouraged catheterization laboratory technicians to expand their skills and capabilities, fostering the teamwork across professions. Additional prep time for the rPCI platform and procedural steps must be considered, leading to prolonged procedures compared to manual PCI [11, 17]. Sufficient staff training and the implementation of procedural shortcuts (e.g. pre-loading of PCI balloons) helped to streamline rPCI cases. These steps reduced the additional prep time to a reasonable amount in our experience but further refinements should address this aspect.

Economic considerations are highly dependent on national healthcare settings. In Germany, additional costs for the acquisition of the rPCI platform and the purchase of the disposable cassettes are currently not sufficiently covered by reimbursement. This aspect may limit the broad dissemination of the technology among in certain healthcare settings. On the other hand, rPCI has shown to reduce occupational hazards and minimize radiation exposure for the interventionalists [9], while these results were conflicting regarding patients’ exposure [17, 18]. In the current study, the dose area product was lower compared to other rPCI cohorts [11, 19, 20], although procedural and fluoroscopy times were in a similar range in the majority of cases [11, 19]. In our opinion, these findings cannot be explained by the use of the robotic platform itself rather than a strong focus on radiation protection at our institution as published before [2]. Additionally, dose area products for manual PCIs at our institution were in the same range compared with the presented rPCI data [2], whereas others found the dose area products of rPCI to be lower compared to manual PCI [11, 20]. Randomized studies a required to investigate the direct comparison of robotic versus manual PCI with regard to procedural parameters and the radiation exposure of the patient and to explore the long-term effects of these aspects weighed against the additional resources required for rPCI.

All procedures were completed successfully which was in accordance with high success rates reported by others [9, 11, 20]. Most simple lesions were exclusively treated robotic-assisted. With higher lesion complexity, the rate of manual assistance increased. This was largely driven by planned manual maneuvers (e.g. wire passage in CTO lesions or “hybrid approach” in bifurcation stenting). Unplanned manual assistance was employed occasionally, mainly for difficulties with guiding catheter stabilization or backup [19]. Conflicting rates of manual assistance [11, 19, 20] may be explained by the high rate of complex and CTO lesions in our series. Others had excluded CTO lesions [20], planned two-stent strategies and severely calcified stenoses from their analyses [9, 11, 19, 20]. After a short learning curve, we intentionally integrated manual steps into complex procedures to expedite rPCI and combine benefits of both techniques (“hybrid approach”), as discussed below. This may have led to lower rates of conversion to manual PCI in our series as compared to others [11, 20].

Procedural myocardial infarction according to the SCAI classification was observed in four cases. None of these patients were symptomatic or had further clinical evidence of myocardial damage. The diagnoses were solely based on increased values of high-sensitivity troponin I [16]. Distinct endpoint definitions, cutoff values, and lesion complexity may be responsible for the relevant number of periprocedural myocardial infarctions reported in this series [9, 11, 20]. One non-disabling stroke occurred after complex manual-assisted rPCI of a venous bypass graft. Whether this was linked to procedural aspects or the patient’s risk profile remains unclear.

We found automated wire movements (technIQ™) to be very helpful, particularly in crossing complex lesions and engaging angulated side branches. It allowed for precise and predictable movements of the wire tip and was hence used in most procedures, often as a combination of several automated movements. Whether this feature may particularly aid inexperienced interventionalists in crossing complex lesions remains to be determined. To the best of our knowledge, there are no time measurements available in the literature precluding a direct comparison of wiring times as well as potential advantages of automated wire movements in the current study. But it demonstrates the potential in this field and rPCI-facilitated autonomous wire advancement to a defined target according to a three-dimensional imaging dataset may not be too far away. One current limitation relates to the lack of tactile feedback. This is of importance for wire crossing in CTO lesions or delivery of catheters in long and calcified lesions, particularly with higher profile devices, e.g. cutting balloons, intravascular lithotripsy or intravascular ultrasound catheters. To visualize the force necessary to advance a certain device may be beneficial to adjust and adapt procedural steps during rPCI, as this was the major driver for conversion to manual PCI in our experience. The intentional combination of manual steps and robotic-assisted movements proved beneficial in certain scenarios and to expedite complex procedures. This “hybrid approach” facilitated the simultaneous control over two delivery systems (e.g. two balloons for kissing-balloon maneuver or balloon and stent). Future developments of the rPCI platform should address the potential to steer at least two coronary wires and two monorail delivery systems simultaneously. This may further ease and expedite robotic procedures.

rPCI may be performed remote over long distances, as successfully demonstrated before [10, 21]. This may prove particularly useful in underserved areas or in pandemic scenarios [22]. Whether rPCI systematically improves precision PCI through optimized sizing or prevention of geographic mismatch has yet to be determined. Nevertheless, integrating multimodality imaging and physiology into a cockpit solution may significantly enhance PCI workflows.

Limitations of our analysis relate to the single-arm single-center design impeding direct comparison with manual PCI. Our focus was to assess clinically meaningful results and evaluate potential benefits and challenges establishing an rPCI program in clinical routine. As a reduction of radiation exposure had been demonstrated repeatedly for rPCI, we refrained from additional measurements of radiation doses in this project and focused on specific technical aspects and clinical outcomes.

## Conclusion

The use of rPCI was feasible and safe in clinical routine even in complex coronary lesions. In our experience, interventional cardiologists had a short learning curve adjusting to the current system. Moreover, the application of rPCI fostered teamwork across professions in the cathlab team. Nevertheless, several challenges need to be addressed before broad application of rPCI. These include technical refinements of the robotic platform, integration of advanced imaging to facilitate robotic (rather than robotic-assisted) PCI, and economic aspects. The “sweet spot “ for rPCI at which the benefits of robotic-assisted vs. manual PCI outweigh the additional resources currently required has yet to be defined and randomized controlled trials are eagerly awaited to address this question. Nevertheless, the latest generation rPCI platform offers a glimpse into what the future of PCI may look like if the platform is continuously and rigorously refined.
